# Age-Related Enhancements in Positive Emotionality across The Life Span: Structural Equation Modeling of Brain and Behavior

**DOI:** 10.1523/JNEUROSCI.1453-21.2022

**Published:** 2022-04-20

**Authors:** Jason Stretton, Susanne Schweizer, Tim Dalgleish

**Affiliations:** ^1^Medical Research Council, Cognition and Brain Sciences Unit, University of Cambridge, Cambridge CB2 7EF, United Kingdom; ^2^Department of Psychology, University of New South Wales, Sydney, New South Wales 2052 Australia; ^3^Cambridgeshire and Peterborough National Health Service Foundation Trust, Cambridge CB21 5EF, United Kingdom; ^4^Cambridge Centre for Ageing and Neuroscience, University of Cambridge, Cambridge CB2 1TN, United Kingdom

**Keywords:** aging, emotion, emotion regulation, positivity, structural equation modeling

## Abstract

Aging is associated with a bias in attention and memories toward positive and away from negative emotional content. In addition, emotion regulation appears to improve with age, despite concomitant widespread cognitive decline coupled with gray matter volume loss in cortical and subcortical regions thought to subserve emotion regulation. Here, we address this emotion-aging paradox using the behavioral data of an emotion regulation task from a population-derived, male and female, human sample (CamCAN) and use structural equation modeling together with multivariate analysis of structural MRI images of the same sample to investigate brain–behavior relationships. In a series of measurement models, we show the relationship between age and emotionality is best explained by a four-factor model, compared with single and hierarchical factor models. These four latent factors are interpreted as Basal Negative Affect, Positive Reactivity, Negative Reactivity and Positive Regulation (upregulating positive emotion to negative content). Increasing age uniquely contributes to increased Basal Negative Affect, Positive Reactivity, and Positive Regulation, but not Negative Reactivity. Furthermore, we show gray matter volumes, namely in the bilateral frontal operculum, medial frontal gyrus, bilateral hippocampal complex, bilateral middle temporal gyri, and bilateral angular gyrus, are distinctly related to these four latent factors. Finally, we show that a subset of these brain–behavior relationships remain significant when accounting for age and demographic data. Our results support the notion of an age-related increase in positivity and are interpreted in the context of the socioemotional selectivity theory of improved emotion regulation in older age.

**SIGNIFICANCE STATEMENT** Aging is associated with a paradoxical increase in well-being and improved emotion regulation despite widespread cognitive decline and gray matter volume loss in neural regions that underlie emotion regulation. Using a population-derived sample, we test the theories behind this emotion/aging paradox with an emotion regulation task and structural MRI data. We report robust age-related increases in positivity across the life span and show structural neural integrity influences this relationship with increasing age. Several brain–behavior relationships remained unaffected by age and may represent empirically derived neural markers to explore the paradox of increased well-being in old age. The results support the predictions of socioemotional selectivity theory of improved emotion regulation in older age and challenge the amygdala-focused neural predictions of the aging brain model.

## Introduction

Current reported levels of personal well-being in the United Kingdom are at their highest after the age of 65 (https://www.ons.gov.uk/peoplepopulationandcommunity/wellbeing/articles/measuringnationalwellbeing/atwhatageispersonalwellbeingthehighest). Although ratings do fall from age 75 onward, epidemiological surveys of self-reported life satisfaction and happiness in those over 90 remains higher than that of middle-aged individuals (https://www.ons.gov.uk/peoplepopulationandcommunity/wellbeing/articles/measuringnationalwellbeing/atwhatageispersonalwellbeingthehighest). This is supported by laboratory studies that reveal that healthy older adults report enhanced positive affect and greater emotional stability than their younger counterparts (Carstensen et al., 2000, 2011). Given the ubiquity of physical and cognitive decline associated with getting older, it is somewhat paradoxical that our twilight years are often the source of greatest satisfaction.

Socioemotional selectivity theory (SST; Carstensen, 1992, 2006), an influential life span theory of motivation, seeks to unravel this paradox. Specifically, SST suggests that adult life is governed by a core sets of goals broadly associated with either the acquisition of knowledge, resources, and social connections or the regulation of emotions associated with well-being. The central tenet of SST is that the relative importance of these sets of goals changes as a function of future time horizons. When the future is perceived as lengthy, as is typical in youth, goals associated with acquisition to maximize future prospects are prioritized over those associated with more immediate positive emotionality and well-being. Conversely, when the future is perceived as constrained, typically as we get older, our goal priorities shift to become less future oriented and more focused on the emotional satisfaction that is possible in the here and now. Other prominent theories in this domain similarly emphasize age-related shifts in strategic priorities and consequent behavioral preferences (Labouvie-Vief, 2003; Charles, 2010; Urry and Gross, 2010).

A related theoretical view is that the greater emotional stability and well-being in older adults might also be a function of age-related increases in expertise in navigating emotive situations, either through more optimal selection of appropriate emotional regulation strategies or as a function of more effective and consolidated emotion-control skills (John and Gross, 2004; Birditt et al., 2005; Sims et al., 2015; Burr et al., 2020). Support for this latter view is mixed, however. Some studies are consistent, whereas others show no age-related changes in emotion regulation or declines in regulation capacity with age (Phillips et al., 2008; Urry and Gross, 2010; Winecoff et al., 2011; Opitz et al., 2014; Sims et al., 2015; Martins et al., 2018; Livingstone and Isaacowitz, 2019; Schweizer et al., 2019; Burr et al., 2020). One reason for these mixed data might be the reliance of several core emotion regulation strategies—for example, reappraisal—on domain-general processes of cognitive control (Ochsner and Gross, 2005), which appear to decline with age as a function of sharp neuronal degradation of the dorsolateral prefrontal cortex (Braver and Barch, 2002).

However, differential structural trajectories of the subregions of the PFC may help to explain why older adults maintain function in other emotion processing contexts such as memory and attention. Prefrontal regions including the ventromedial prefrontal cortex (Quirk and Beer, 2006; Winecoff et al., 2013) and anterior cingulate cortex (Bush et al., 2000), both associated with emotion processing, have been shown to maintain their cortical thickness across the life span (Fjell et al., 2009a)

A compelling and competing account suggests age-related improvements in emotion regulation are caused by an age-related change in the amygdala response to affective stimuli, the aging brain model (ABM; Cacioppo et al., 2011). The ABM argues that the amygdala maintains its responsiveness to positive stimuli as we age but diminishes in response to negative stimuli, thus biasing the attention to and subsequent memory of positive stimuli. Functional imaging evidence shows older adults have reduced amygdala activity in response to negative, but not positive, pictures, relative to younger adults (Mather et al., 2004), whereas structural imaging and postmortem studies indicate less volumetric decline and less histologic effects of aging in the amygdala as a proxy of better preserved function (Allen et al., 2005; Brabec et al., 2010).

Indeed, prominent theories of emotion regulation draw a distinction between effortful, resource-demanding strategies and processes that have become automatic and thus less reliant on declining cognitive control resources (Braunstein et al., 2017). There is now robust support from laboratory studies that automatic cognitive processing biases in domains such as attention and memory in favor of positive information—the so-called positivity effect—are augmented with older age (Charles et al., 2003; Kennedy et al., 2004; Mather and Carstensen, 2005). It therefore follows that automatized emotion regulation processes (including those that drawn on attentional and mnemonic processes) that prioritize positive affect become strengthened across the life span, whereas regulation processes that rely on declining effortful cognitive control may become less effective. This might help to explain the mixed findings regarding emotion regulation and aging. Another important factor that may partially account for these equivocal data is the tendency for studies to use single bivalence scales (Schweizer et al., 2019), ranging from positive to negative, to measure experienced affect. Single scales could obscure age-related increases in positive affectivity if there are separate age-related effects for negative affectivity that act in an opposite direction. A strength of the positivity effect literature (Charles et al., 2003; Kennedy et al., 2004; Mather and Carstensen, 2005) is the disaggregation of positive and negative information. A similar measurement separation in the domain of valence would be consistent with research in affective neuroscience confirming the utility of discrete valence dimensions (Viinikainen et al., 2010; Paulus et al., 2017).

### Current study

The present study, therefore, used a gold standard laboratory-based emotional reactivity and regulation task (Schweizer et al., 2013, 2016) to examine age-related changes in elicited positive affectivity (assayed independently of negative affectivity) within both passive viewing and emotion regulation contexts. We used a large population-derived sample of adults aged 18–88 as part of the Cambridge Center for Ageing and Neuroscience (Cam-CAN) cohort (https://www.cam-can.org; Shafto et al., 2014). We evaluated the hypothesis that the robust age-related positivity effect within the cognitive domain extends to the affective domain in the form of an age-dependent increase in core components of positive affectivity. We predicted that such enhancement in positive affectivity would be independent of any age-related changes in cognitive control measured behaviorally. Finally, we examined the relationships between age-related effects in positive affectivity and age-related differences in brain gray matter (GM), based on the magnetic resonance (MR) measurements within the Cam-CAN cohort (Taylor et al., 2017). This allowed us to evaluate whether any age-related enhancements in positive affectivity were also independent of age-related volumetric changes in frontoparietal brain regions typically associated with more effortful emotion regulation (Fjell et al., 2009b) using structural equation modeling to explore brain–behavior relationships (Kievit et al., 2012).

## Materials and Methods

### Participants

Three hundred and thirty individuals of the Cam-CAN (Shafto et al., 2014) sample were invited to perform an emotion reactivity and regulation task. Sixteen participants chose not to undertake the task, and 26 participants were excluded as session notes indicated they did not follow the instructions of the task appropriately. Fifteen participants did not complete our measure of cognitive control, the Cattell Test (*n* = 9), nor provide demographic data (*n* = 6), and for an additional 24 there were problems with the analysis of their MR images (Taylor et al., 2017). Demographic data for the remaining 249 are shown in Table 1. All participants took part in a range of psychological tests (No other cognitive domains were analyzed in the context of this analysis.). Ethical approval for the study was obtained from the Cambridgeshire 2 (now called East of England—Cambridge Central) Research Ethics Committee. Participants gave full informed consent.

**Table 1.** Sample demographics

Decade	1	2	3	4	5	6	7
*n*	27	38	44	36	40	39	25
Age range; M (SD)	18–28; 24.1 (3.6)	29–38; 33.8 (2.6)	39–48; 44.6 (2.9)	49–58; 53.7 (2.9)	59–68; 64.1 (2.7)	69–88; 73.4 (3.1)	79–88; 82.3 (2.8)
Female *n* (%)	16 (59)	19 (50)	21 (48)	21 (58)	19 (47)	22 (56)	13 (52)
Education *n* (%)							
None		1 (3)	3 (7)	1 (3)	3 (7)	5 (13)	4 (16)
GCSE	5 (18.5)	3 (8)	7 (16)	6 (17)	13 (33)	9 (23)	5 (20)
A-level	5 (18.5)	5 (13)		9 (25)	4 (10)	4 (10)	4 (16)
University degree	17 (63)	29 (76)	34 (77)	20 (55)	20 (50)	21 (54)	12 (48)
Fluid intelligence M (SD)	37.3 (4.7)	37.5 (3.6)	35.8 (3.7)	33.4 (4.2)	30.9 (4.7)	27.7 (5.7)	24.9 (3.5)

M, Mean.

### Experimental design

#### Emotion reactivity and regulation task

**Figure 1.**
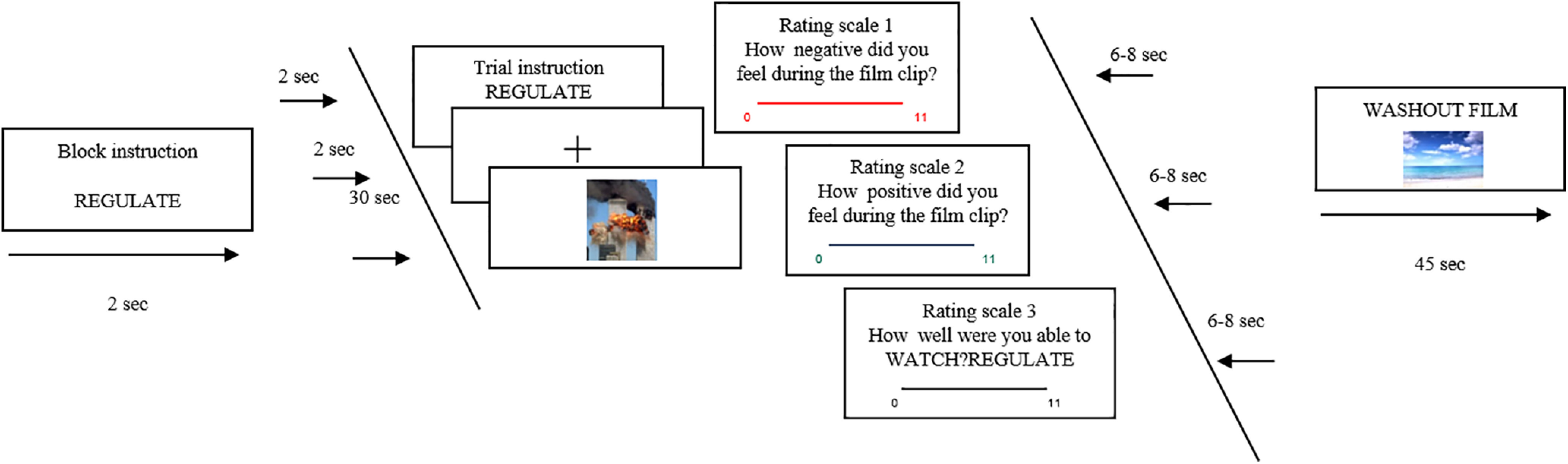
A sample trial of the Emotion Reactivity and Regulation Task in which participants viewed a set of negative, neutral, or positive 30 s film clips. In the WATCH condition, participants were asked to simply watch the films and allow their emotions to arise naturally. In the REGULATE condition (example in the figure), participants were asked to reappraise their emotions to a negative film by changing the way they thought about the content of the film. After each film clip, participants rated the positive and negative affects that they felt during the film on a two valence scale, as well as their compliance with the task instructions.

Emotion reactivity and regulation were assessed with a film-based paradigm (Schweizer et al., 2013; 2016; Fig. 1). Participants viewed a series of 40 film clips of 30 s that were either positive (e.g., infants laughing), neutral (e.g., weather report), or negative (e.g., documentary of the Rwandan genocide) in valence and consisted of a mixture of real-life and fictional footage. Participants received one of two different viewing instructions before each clip, either (1) WATCH, where participants were told to watch the film clips and allow themselves to feel any emotions that naturally arose without trying to deliberately distract themselves from the content of the film clip or effortfully regulate their emotions in any way, or (2) REGULATE, which was only applicable to half of the negative film clips. Here, participants were explicitly asked to try to reduce (downregulate) any unwanted distressing affect in response to the film clip by reappraising the contents of the film clip. This gave four task conditions: Positive Watch, Negative Watch, Negative Regulate, and Neutral Watch.

Before each film clip, participants received a prompt to indicate the valence and viewing instruction for the clip (e.g., WATCH NEUTRAL or REGULATE NEGATIVE). This was followed by the clip itself, after which participants rated on separate 10-point scales both their negative and positive affective reactions experienced during the clip, as well as rating how much they simply watched the clip versus regulated their affect as a measure of compliance. This resulted in both mean negative and mean positive affective ratings for each task condition in each participant. Each of the four conditions yielded a positive affect rating and a negative affect rating. Affective responses were rated on a scale ranging from 1 (not at all), to 11 (extremely). Instruction compliance was rated on a scale ranging from 1 (Watch) to 11 (Regulate). Therefore, eight ratings were used for modeling purposes to disaggregate the positive and negative affective ratings.

Instruction, film clip, and affective and compliance ratings together composed an experimental trial. Each condition was presented twice with four trials in each block. Emotional blocks were followed by 45 s washout clips, that is, a calming film clip (e.g., waves gently rolling back and forth on a beach with a meditative soundtrack), to return affective levels to prestimulus baseline. Films were randomized across the WATCH and REGULATE conditions separately for each participant, and the presentation order of condition was pseudo randomized, always starting with a neutral block and ending with a positive block.

### Net emotional effects of age

The standard method of analysis for behavioral data of emotion regulation tasks is to subtract the raw emotion score (scaled from negative to positive) from the neutral stimulus from the raw emotion score from the affective stimulus (positive or negative trials) to create positive and negative reactivity indices. In our task, we challenged affective response after each stimulus on separate positive and negative scales (ranging from not at all to extremely). To examine the standard response we recreated these scales accounting for the residual emotionality for each scale and regressed them against age to determine age-related changes in net emotionality. In each participant, we first removed the residual positivity/negativity associated with each scale. To do this, we subtracted the positivity/negativity associated with watching positive/negative films from the positivity/negativity scale associated with watching positive/negative films. We then did the same for the Neutral film clips (subtracted negative from positive scales). To gain a net effect score, we then subtracted the Neutral score from either the Positive (for Positive Reactivity) or Negative (for Negative Reactivity) scores as in the following example:

$$\text{Net Positive Reactivity }=\left({\text{Positive}}^{\text{Watch}}{}_{\text{positive}}–{\text{ Positive}}^{\text{Watch}}{}_{\text{negative}}\right)–({\text{Neutral}}^{\text{Watch}}{}_{\text{positive}}–{\text{Neutral}}^{\text{Watch}}{}_{\text{negative}})$$

$$\text{Net Negative Reactivity }=\left({\text{Negative}}^{\text{Watch}}{}_{\text{negative}}–{\text{ Negative}}^{\text{Watch}}{}_{\text{positive}}\right)–({\text{Neutral}}^{\text{Watch}}{}_{\text{negative}}–{\text{Neutral}}^{\text{Watch}}{}_{\text{positive}})$$

$$\text{Net Negative Regulation }=\left({\text{Negative}}^{\text{Regulate}}{}_{\text{negative}}–{\text{ Negative}}^{\text{Regulate}}{}_{\text{positive}}\right)–({\text{Negative}}^{\text{Watch}}{}_{\text{negative}}–{\text{Negative}}^{\text{Watch}}{}_{\text{positive}}).$$

### Demographic, cognitive, and mental health measures

Baseline characteristics collected from the Cam-CAN cohort include age, sex, history of depression (yes/no), and a self-reported measure of highest level of education obtained, scored from the following: (1) Basic (e.g., left education before the age of 16), (2) General Certificate of Secondary Education (GCSE)/O-level (e.g., left education before the age of 18), (3) A-level (e.g., left education after the age of 18), and (4) Degree (e.g., left university after the age of 21 or older). Cognitive control as indexed by fluid intelligence (Duncan, 1995) was measured by the Cattell Culture Fair Test (Scale 2, Form A), administered using pencil and paper according to the standard protocol.

### MRI data, source-based morphometry

Shafto et al. (2014) provide details of the MRI sequences, and Taylor et al. (2017) provide details of the MRI preprocessing. Voxel-based morphometry is a univariate method and does not use any information about the relationships among voxels. In addition, it will only detect voxels for which a specific predicted effect is present (typically, a mean difference between two groups). In contrast, a multivariate, data-driven approach can provide a way to pool information across different voxels as well as identify unpredicted patterns. The voxels that carry similar information will group to a set of regions. Source-based morphometry (SBM) uses independent component analysis (ICA) to extract maximal spatially independent sources revealing patterns of variation that occur in structural MRI images (Xu et al., 2009).

Before ICA, we estimated the number of components for extraction using an information theoretic approach. First, we uniformly subsampled the gray matter (GM) images until the estimated entropy rate equaled the entropy rate of an independent and identically distributed Gaussian random process of the same variance and data length. Next, we estimated the number of components using the Akaike's Information Criterion (AIC) resulting in 51 estimated components.

All gray matter images were processed using spatial ICA (Calhoun et al., 2001) as implemented in the GIFT toolbox (http://icatb.sourceforge.net). ICA was performed using a neural network algorithm (infomax) that attempts to minimize the mutual information of the network outputs (Bell and Sejnowski, 1995). Every gray matter image is converted into a one-dimensional vector. The 249 gray matter images of each participant were arrayed into one 249-row, subject-by-gray-matter data matrix. This matrix was then decomposed into a mixing matrix and a source matrix. The mixing matrix expresses the relationship between 249 subjects and *k* (*n* = 51) components. The rows of the matrix are scores that indicate to what degree the *k* components contribute to a given subject. The columns of the matrix indicate how one component contributes to the 249 subjects. In contrast, the source matrix expresses the relationship between the *k* components and the voxels within the brain. The rows of the matrix indicate how one component contributes to different brain voxels, and the columns of the matrix are scores that indicate how one voxel contributes to each of the components. We used the source matrix for visualization. We reshaped every row of the source matrix back into a 3D image (source map). These source maps were scaled to unit SD (SBM *Z* map) and thresholded at a value of |*Z*| > 3:0. The maps of the sources were then superimposed on the Montreal Neurological Institute–normalized template brain.

We selected 12 bilaterally represented components that anatomically (Harvard-Oxford Atlas) overlapped with emotion-reactivity- and regulation-related regions taken from the existing functional imaging literature (Phan et al., 2002; Diekhof et al., 2011; Buhle et al., 2014; Frank et al., 2014; Kohn et al., 2014); precuneus/posterior cingulate cortex (PCC), superior frontal gyrus (SFG), middle temporal gyrus (MTG), anterior/middle cingulate cortex (A/MCC), middle frontal gyrus (MFG), inferior frontal gyrus (IFG) pars triangularis, IFG pars opercularis, ventral striatum, hippocampus/amygdala complex, medial frontal gyrus, anterior insula, and angular gyrus. Data extracted from the mixing matrix for each source were entered into the structural equation model (see Fig. 4, see Table 3, visualization and covariance matrix).

### Statistical analysis

Analysis of the effects of age was performed by multiple regression within general linear models (GLMs) that treated age as a continuous variable modeled by linear and quadratic terms. We focus on effect sizes (*R*^2^), expressed as the percentage of variance explained by a specific statistical contrast within the GLM, rather than *p* values as the latter become less appropriate for larger samples.

### Structural equation models

Structural equation models were fit using the package Lavaan71 (Rosseel, 2012) in R version 3.1.2 (https://www.r-project.org/). We used the following guidelines for judging good fit (Schermelleh-Engel et al., 2003): root mean square error of approximation (RMSEA) below 0.05 (acceptable: 0.05–0.08) and a comparative fit index (CFI) above 0.97 (acceptable: 0.95–0.97). All models were fit using maximum likelihood estimation using robust SEs, for which we report the Satorra-Bentler (SB) scaled test statistic; *p* values < 0.05 were used to judge significance of individual paths. We initially tested a single-factor model to establish whether a single general emotionality factor could explain the individual difference in affect ratings. We then tested a four-factor measurement model based on theoretically derived notions of emotional reactivity and regulation. Once the measurement model was established, we finally tested a hierarchical model to assess whether the four factors were influenced by a single general emotionality factor.

## Results

### Emotion reactivity and regulation measurement models

Using confirmatory factor analysis, we tested a series of models that could account for the individual differences in the emotion reactivity and regulation task (ERRT) ratings. A single factor model, with one emotion factor explaining all eight ERRT ratings (NEUTRAL WATCH, *Positive* and *Negative* ratings; POSITIVE WATCH, *Positive* and *Negative* ratings; NEGATIVE WATCH, *Positive* and *Negative* ratings; NEGATIVE REGULATE, *Positive* and *Negative* ratings) did not fit the data well (Table 2). We therefore explored a four-factor model, with each factor hypothetically representative of the theoretically plausible constructs of emotion reactivity and regulation as follows: *Negative Regulation*, reflecting decreases in negative affectivity in response to negative stimuli and consequently loading negatively on NEGATIVE REGULATE *negative* ratings and NEGATIVE WATCH *negative* ratings; Positive Regulation, reflecting increased positive affectivity in response to negative stimuli and consequently loading positively on NEGATIVE REGULATE positive ratings and NEGATIVE WATCH positive ratings; *Negative Reactivity*, reflecting acute increases in negative affectivity and consequently loading on *negative* ratings across all three WATCH conditions; and *Positive Reactivity*, loading on the *positive* ratings from the POSITIVE and NEUTRAL WATCH conditions. This four-factor model fit the data significantly better than the single-factor model (Δχ2 = 192, Δdf = 8, *p* = 2.2e-16), but remained just below the predefined threshold for good fit (Table 2).

**Table 2.** Fits of the main structural equation models tested

Model	χ2	df	RMSEA	CFI	SB	AIC
Single factor	305	20	0.24	0.69	1.40	5084
Hierarchical factor	DNC	DNC	DNC	DNC	DNC	DNC
Four factor	88	12	0.16	0.92	1.22	4781
Four factor modified	11	9	0.034	0.99	1.25	4694
Four factor modified with age	17	13	0.038	0.99	1.30	6747
Four factor with age, education, and IQ	52	22	0.075	0.97	1.20	8007
Four factor with age, education, and IQ*	65	34	0.061	0.97	1.11	9397
Four factor with GM and education*	79	69	0.024	0.99	1.01	13550
Four factor with GM and age*	93	87	0.017	0.99	1.02	15469

DNC, Did not converge; IQ, intelligence quotient.

*Indicates these models additionally contained Gender and Depression variables (affecting all latent factors). Note that the χ^2^ reported is the Satorra-Bentler scaled χ2, with the scaling factor reported as SB.

**Table 3.** Correlation matrix of source-based morphometry sources

Correlations												
		Precuneus/posterior cingulate cortex	Superior frontal gyrus	Middle temporal gyrus	Anterior/ middle cingulate	Middle frontal gyrus	Inferior frontal gyrus; pars triangularis	Ventral striatum	Hippocampal/ amgydala complex	Medial frontal gyrus	Anterior insula	Inferior frontal gyrus, operculum
Superior frontal gyrus	Pearson's correlation	0.371**										
	Sig. (two tailed)	0.000										
	*N*	249										
Middle temporal gyrus	Pearson's correlation	0.620**	0.381**									
	Sig. (two tailed)	0.000	0.000									
	*N*	249	249									
Anterior/middle cingulate	Pearson's correlation	0.229**	0.141*	0.338**								
	Sig. (two tailed)	0.000	0.027	0.000								
	*N*	249	249	249								
Middle frontal gyrus	Pearson's correlation	0.518**	0.590**	0.502**	0.244**							
	Sig. (two tailed)	0.000	0.000	0.000	0.000							
	*N*	249	249	249	249							
Inferior frontal gyrus; pars triangularis	Pearson's correlation	0.564**	0.483**	0.564**	0.298**	0.577**						
	Sig. (two tailed)	0.000	0.000	0.000	0.000	0.000						
	*N*	249	249	249	249	249						
Ventral striatum	Pearson's correlation	0.563**	0.457**	0.579**	0.264**	0.532**	0.536**					
	Sig. (two tailed)	0.000	0.000	0.000	0.000	0.000	0.000					
	*N*	249	249	249	249	249	249					
Hippocampal/amygdala complex	Pearson's correlation	0.569**	0.306**	0.659**	0.322**	0.462**	0.523**	0.531**				
	Sig. (two tailed)	0.000	0.000	0.000	0.000	0.000	0.000	0.000				
	*N*	249	249	249	249	249	249	249				
Medial frontal gyrus	Pearson's correlation	0.647**	0.453**	0.706**	0.253**	0.634**	0.667**	0.624**	0.615**			
	Sig. (two tailed)	0.000	0.000	0.000	0.000	0.000	0.000	0.000	0.000			
	*N*	249	249	249	249	249	249	249	249			
Anterior insula	Pearson's correlation	0.584**	0.422**	0.623**	0.358**	0.495**	0.618**	0.601**	0.569**	0.688**		
	Sig. (two tailed)	0.000	0.000	0.000	0.000	0.000	0.000	0.000	0.000	0.000		
	*N*	249	249	249	249	249	249	249	249	249		
Inferior frontal gyrus, operculum	Pearson's correlation	0.494**	0.291**	0.578**	0.246**	0.442**	0.470**	0.391**	0.503**	0.597**	0.523**	
	Sig. (two tailed)	0.000	0.000	0.000	0.000	0.000	0.000	0.000	0.000	0.000	0.000	
	*N*	249	249	249	249	249	249	249	249	249	249	
Angular gyrus	Pearson's correlation	0.284**	0.074	0.327**	0.094	0.189**	0.188**	0.161*	0.280**	0.284**	0.230**	0.186**
	Sig. (two tailed)	0.000	0.246	0.000	0.141	0.003	0.003	0.011	0.000	0.000	0.000	0.003
	N	249	249	249	249	249	249	249	249	249	249	249

**Correlation is significant at the 0.01 level (two tailed).

*Correlation is significant at the 0.05 level (two tailed). Sig. = significant.

We next investigated the modification indices of the four-factor model to ask whether freeing any parameters would improve model fit. The value of a given modification index (m.i.) is the minimum amount that the χ^2^ statistic is expected to decrease if the corresponding parameter is freed. This suggested the following three minor modifications to the model: (1) to account for the residual anticorrelation between *positive* and *negative* emotion ratings elucidated from watching positive content (m.i. = 42.41), (2) to account for the anticorrelation between positive emotion ratings from the POSITIVE WATCH condition and the latent factor of Negative Reactivity (derived from all negative emotion ratings from the Watch conditions (m.i. = 24.08); and (3) to account for the residual covariance associated with negative emotion ratings from the NEGATIVE REGULATE condition and positive emotion ratings from the NEGATIVE WATCH condition, that is, the assertion that regulation of negative content is associated with the degree of positivity derived from simply watching negative content (m.i. = 18.26). With these three modifications, the model fit very well (Table 2). Finally, we tested a hierarchical factor model, whereby the four factors of the previous model underlie a single hierarchical factor of general emotionality. This model did not converge and was rejected.

Having established a four-factor measurement model, we then explored the significant individual path loadings for each latent factor to refine our interpretation in line with the theoretical and empirical literature. Starting with the positive affect components, our putative Positive Reactivity factor did load significantly and positively, as anticipated, onto the NEUTRAL WATCH *positive* ratings (0.82) and POSITIVE WATCH *positive* ratings (1.02), and so we retained the interpretation of this factor as an index of *Positive Reactivity*, that is, the degree of positivity derived from both neutral and positive content. Our putative *Positive Regulation* factor loaded positively onto *positive* scores from both the NEGATIVE REGULATE (0.89) and NEGATIVE WATCH (0.77) conditions, representing the degree of positivity extracted from negative content and thus we also kept our interpretation of this latent factor as a form of *Positive Regulation*.

In terms of the negative emotion components, both the NEGATIVE REGULATE *negative* ratings (0.86) and NEGATIVE WATCH *negative* ratings (0.86) loaded comparably strongly but positively rather than negatively onto the latent factor we initially termed *Negative Regulation*. A paired *t* test on the raw scores revealed no significant difference between *negative* emotion ratings when either regulating or simply watching negative content (*t* = 0.057, *p* = 0.954). This, together with the direction of associations, indicated that this latent factor is better interpreted as a measure of *Negative Reactivity* to negative content. Finally, the *negative* ratings from the NEUTRAL WATCH (0.90) and POSITIVE WATCH (0.58) conditions, but importantly not the *negative* ratings from the NEGATIVE WATCH condition (0.06), showed significant loadings on our original *Negative Reactivity* factor. With the strongest relationship to the negative rating from the neutral content, and moderate relationship with deriving negativity from positive films, we deemed that this initially characterized *Negative Reactivity* factor better represents *Basal Negative Affect*, particularly as there was no relationship with *negative* scores in the NEGATIVE WATCH condition.

### Effects of age

**Figure 2.**
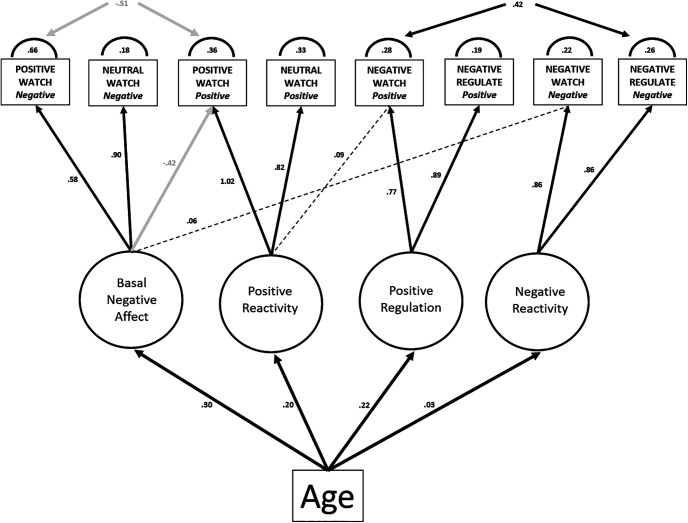
Modified four-factor measurement model and the effects of age. Black paths indicate positive associations, gray paths indicate negative associations, and dashed lines indicate nonsignificant associations.

We next included age as a common cause of individual differences in each latent factor, which produced an excellent overall model fit (Table 2) with parameters shown in Figure 2. Age had a significant relationship with all four latent factors, with factor strength (i.e., higher affect ratings) increasing with increasing age [*Positive Regulation* (*F*_(1,247)_ = 72.5, *p* < 0.0001, *r*^2^ = 0.22; 95% CI = 0.14, 0.31); *Positive Reactivity* (*F*_(1,247)_ = 62.9, *p* < 0.0001, *r*^2^ = 0.20; 95% CI = 0.12, 0.29); *Basal Negative Affect* (*F*_(1,247)_ = 109.3, *p* < 0.0001, *r*^2^ = 0.30; 95% CI = 0.22, 0.39)], although there was a weaker (although significant) influence on *Negative Reactivity* compared with the other three factors [*Negative Reactivity* (*F*_(1,247)_ = 8.1, *p* = 0.004, *r*^2^ = 0.03; 95% CI = 0.00, 0.08)]. Indeed, this model was better than one in which the age-factor paths were constrained to be equal (Δχ2 = 38, Δdf = 3, *p* < 3.32e-08), suggesting differential effects of age on these emotion factors. This supports our primary hypothesis that the factor constructs of both *Positive Reactivity* and *Positive Regulation* increase with age once you disaggregate emotion components by valence.

### Elucidating the contribution of cognitive control

To explore the contribution of cognitive or executive control, we next introduced our measure of fluid intelligence into the model, along with education level to index potential cohort effects. The model fit remained acceptable (Table 2). As expected, age was significantly negatively associated with education level (−0.21) and fluid intelligence (−0.69), reflecting shifts in education accessibility and policy in the United Kingdom over time. However, importantly neither educational level nor fluid intelligence showed any direct significant effects on our two positive emotion factors—*Positive Reactivity and Positive Regulation*—indicating that the significant increases in positive emotion processing with age are above and beyond any concomitant decline in fluid intelligence or cohort effects. The relationship between age and *Basal Negative Affect* also remained significant. However, the positive influence of age on the *Negative Reactivity* factor was no longer significant when including fluid intelligence and education in the model, suggesting that age-related increases in negative reactivity are a function of age-related declines in cognitive control.

### Examining the effects of gender and depression

To further externally validate the model, we next included two variables that have been reported to reliably influence emotion responding—gender and a history of depression. Based on the prior literature, we anticipated that female gender would be significantly associated with both *Positive* and *Negative Reactivity*, independent of the effects of age (Kring and Gordon, 1998). We also anticipated that a history of depression would be associated with higher *Basal Negative Affect* (Kanske et al., 2012) and potentially with higher *Negative Reactivity*, again independent of age.

**Figure 3.**
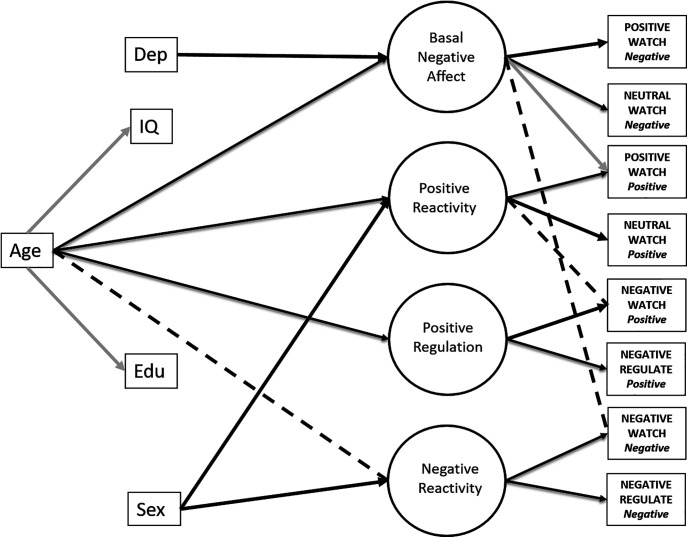
Structural equation model of Age and Emotion including cognitive control (indexed by fluid IQ), education, gender, and depression measures. Age remained significantly positively associated with three latent emotion factors factors when accounting for this additional information. Positive Reactivity and Basal Negative Affect and Positive Regulation all increased with increasing age. Black paths indicate positive associations, gray paths indicate negative associations, and dashed lines indicate nonsignificant associations. Dep, Depression; Edu, educational level; IQ, intelligence quotient. Coefficient weights have been excluded for clarity (see main text).

The model retained its acceptable fit following the inclusion of these variables (Table 2). A history of depression was, as expected, positively associated with *Basal Negative Affect* (0.16) but no other factors. Being female was significantly associated with both *Positive Reactivity* (0.21) and *Negative Reactivity* (0.31) but no other factors. These relationships provide convergent validity for the latent factors in the model. Importantly, the key effects of age on *Positive Reactivity* and *Positive Regulation*, as well as on *Basal Negative Affect*, remained significant beyond the contribution of these additional variables being included in the model (Fig. 3). This is important as other age-related effects on affective processing appear to be a function of age-related differences in depression (Murphy et al., 2019).

### Evaluating the influence of volumetric indices of brain regions involved in emotion reactivity and regulation

**Figure 4.**
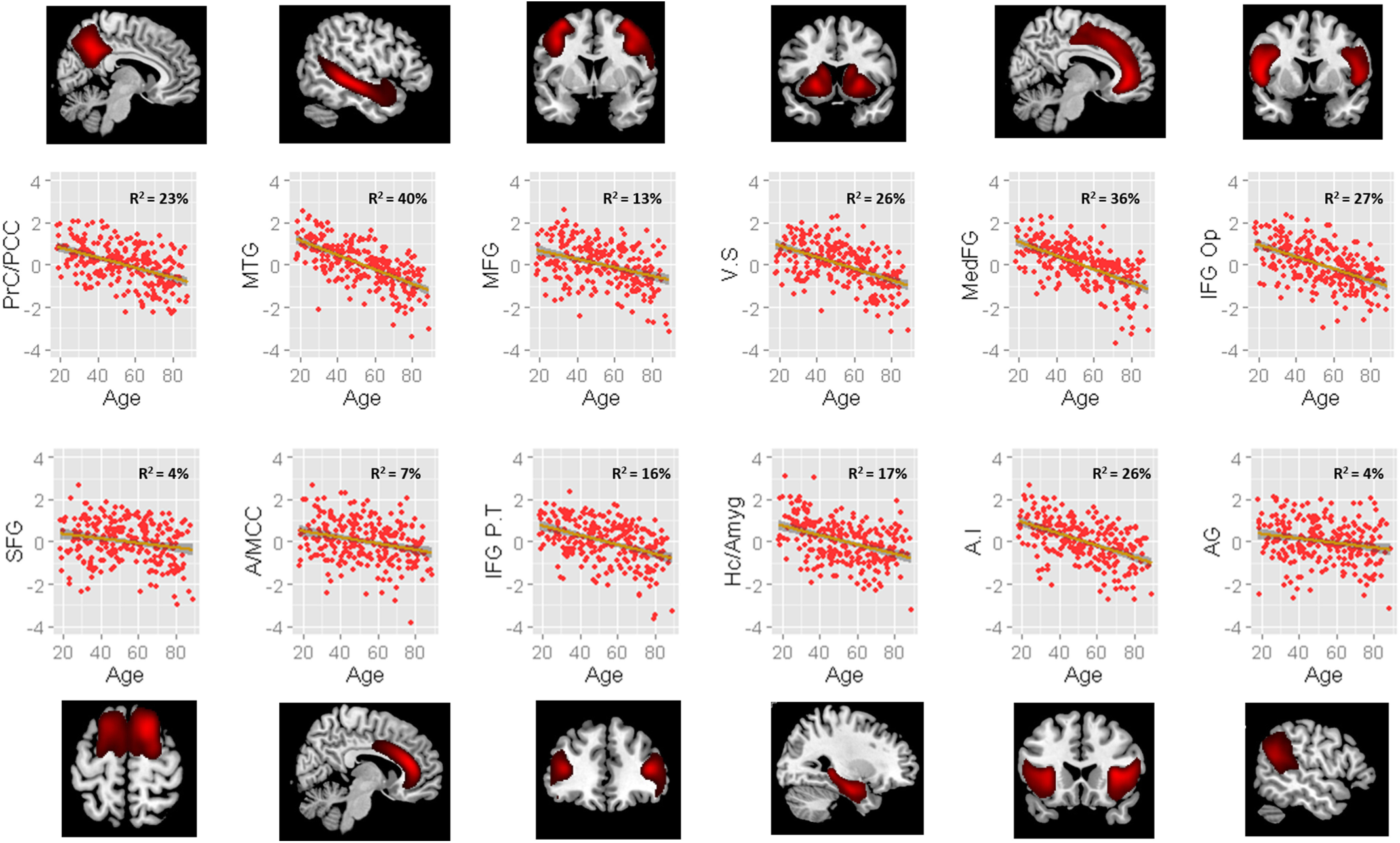
Source-based morphometry sources relationship to age. Each show a linear decline with age. Effect size is shown as *r*^2^. PrC/PCC, Precuneus/posterior cingulate cortex; VS, ventral striatum; MedFG, medial frontal gyrus; IFG Op, inferior frontal gyrus operculum; IFG PT, inferior frontal gyrus pars triangularis; Hc/Amyg, hippocampal complex/amygdala; AI, anterior insula; AG, angular gyrus.

As expected, each of the GM sources declined linearly with age, although with varying degrees (Fig. 4), the MTG showed the greatest effect of age (*r*^2^ = 40%; 95% CI = 0.32, 0.48), whereas the angular gyrus showed the least effect of age (*r*^2^ = 4%; 95% CI = 0.01, 0.10).

We next examined whether there were meaningful relationships between variation in GM in these brain regions and our affectivity factors. Data from the GM sources were included in a new structural equation model as putative causes of the factors. For these models, we retained education, depression, and gender in the model but removed fluid intelligence as here we were, in principle, examining the variance associated with neural indices of cognitive control and did not want these to be obscured by including a behavioral index of cognitive control in the model. It is important to stress that significant paths represent unique covariance between the brain variable and latent variable over and above the shared covariance between the other brain variables. Thus, significant paths should not be interpreted as one-to-one region-behavior mapping. However, model comparison can be used to test the importance of particular combinations of brain variables (Henson et al., 2016).

Model comparison allows us to test the differential partial contribution of each gray matter volume (GMV) source to the different latent factors. First, we imposed equality constraints on all brain–behavior paths to test against the unique contributions of our brain factors. This model failed to converge, suggesting each of the brain–behavior paths need to be estimated freely and that each has unique variability that explains some of the data. We then tested models that zeroed out each GM source to all emotion factors, to investigate the unique contribution of each region to the emotion factors. Independent models showed zeroing out the effect of the PCC (Δχ2 = 2, Δdf = 4, *p* = 0.68), SFG (Δχ2 = 4, Δdf = 4, *p* = 0.45), A/MCC (Δχ2 = 3, Δdf = 4, *p* = 0.62), MFG (Δχ2 = 1, Δdf = 4, *p* = 0.88), pars triangularis (Δχ2 = 5, Δdf = 4, *p* = 0.27), ventral striatum (Δχ2 = 4, Δdf = 4, *p* = 0.44), hippocampus/amygdala (Δχ2 = 6, Δdf = 4, *p* = 0.17), medial frontal gyrus (Δχ2 = 7, Δdf = 4, *p* = 0.12), anterior insula (Δχ2 = 6, Δdf = 4, *p* = 0.21), and angular gyrus (Δχ2 = 5, Δdf = 4, *p* = 0.24) did not significantly change model fit. However, separately setting IFG pars opercularis (Δχ2 = 16, Δdf = 4, *p* < 0.005) and MTG (Δχ2 = 20, Δdf = 4, *p* < 0.001) paths to zero did significantly reduce model fit, indicating a significant independent partial contribution of these regions to the emotion factors.

Five GM sources showed significant paths to our affective factors. Starting with the positive factors, *Positive Reactivity* was negatively associated with GM in the IFG pars opercularis (−0.26). *Positive Regulation* was also negatively associated with GM in IFG pars opercularis (−0.24) and the MTG (−0.42) but positively associated with GM in the hippocampal/amygdala complex (0.19) and angular gyrus (0.16). For the negative emotion factors, *Basal Negative Affect* was also negatively associated with GM in IFG pars opercularis (−0.24) and with GM in the medial frontal gyrus (−0.29). There were no significant brain–behavior relationships between any GM source and the *Negative Reactivity* factor.

### The relationship of age to emotion-related brain–behavior associations

**Figure 5.**
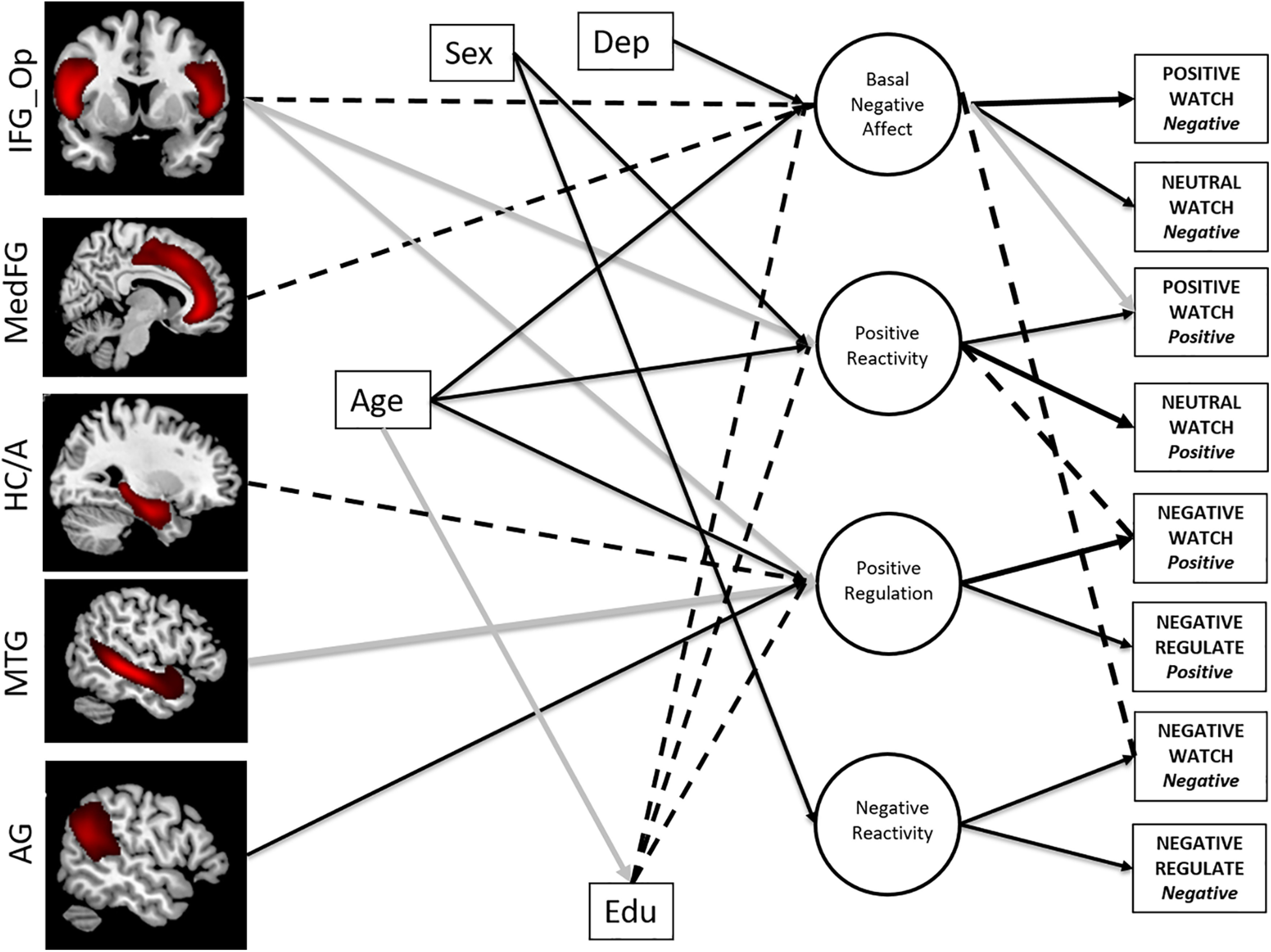
Brain–behavior model with age included. Three paths no longer reach significance; pars opercularis and medial frontal gyrus to Basal Negative Affect, and hippocampal/amygdala complex to Positive Regulation (dashed lines). For clarity, only significant/changed paths are shown. Black paths indicate positive associations, gray paths indicate negative associations, and dashed lines indicate nonsignificant associations. The full model fit is provided in Table 2. Dep, Depression; Edu, educational level, IFG_Op, inferior frontal gyrus pars opercularis; MedFG, medial frontal gyrus; HC/A, hippocampal/amygdala complex; AG, angular gyrus. Coefficient weights have been excluded for clarity (see main text).

We next entered age into the brain–behavior model connected to each of the four emotion factors. Model fit remained excellent (Table 2). Critically, mirroring the findings after adjusting for our behavioral index of cognitive control (fluid intelligence), the positive relationship of age with the two components of positive emotion—*Positive Regulation* and *Positive Reactivity*—remained significant after the brain variables were introduced, indicating that age-related improvements in positive emotion processing are above and beyond any concomitant decline in brain volume in regions associated in the literature with emotion reactivity and regulation. The significant relationship between age and *Basal Negative Affect* was also preserved (Fig. 5).

**Figure 6.**
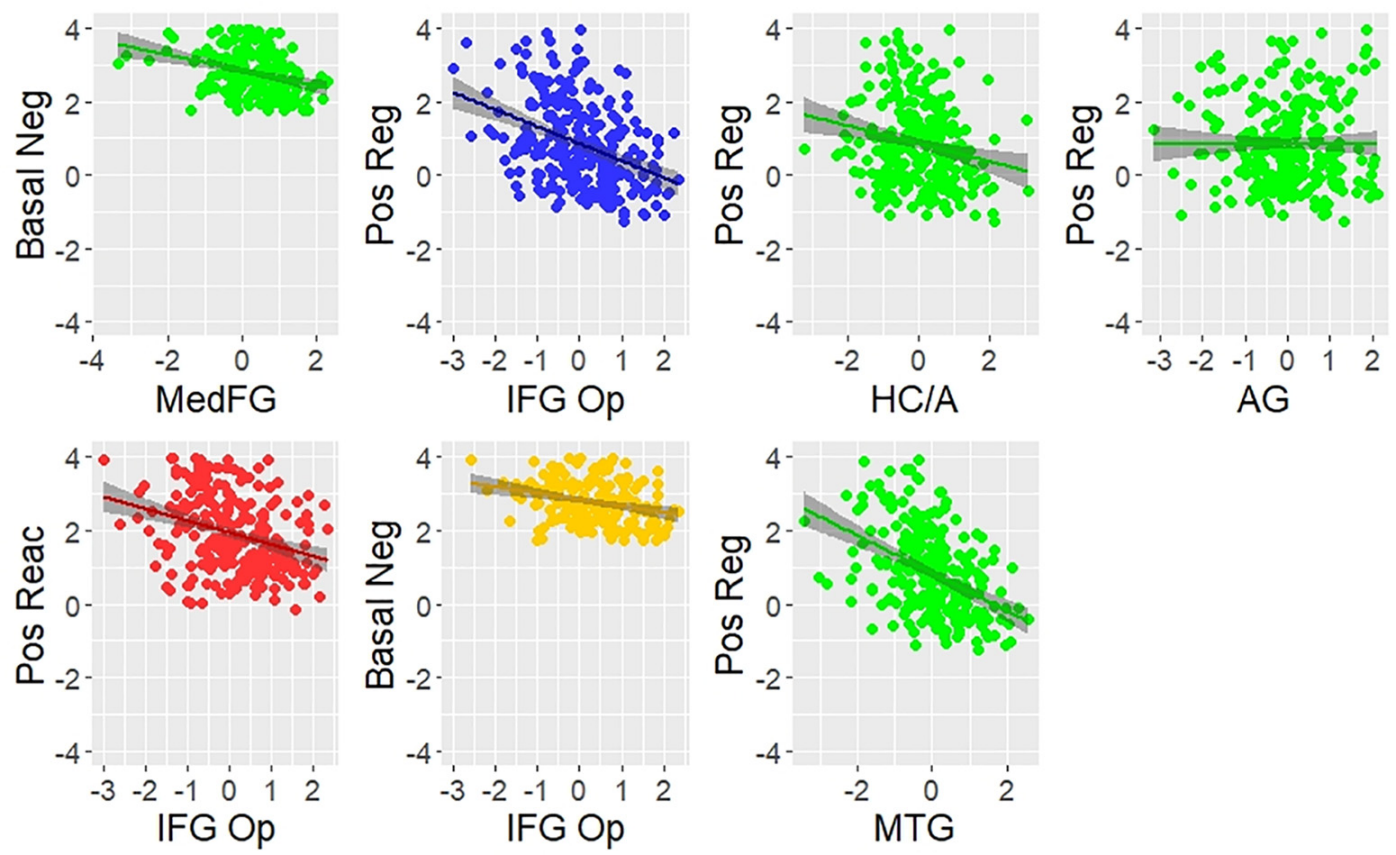
Scatter plots of the brain–behavior pathways in the full model. Pos Reg, Positive regulation; Basal Neg, basal negative affect; Pos Reac, positive reactivity; IFG Op, inferior frontal gyrus pars opercularis; MedFG, medial frontal gyrus; HC/A, hippocampal/amygdala complex; AG, angular gyrus.

Three brain–behavior paths were no longer significant once age was included in the model. The medial FG and IFG pars opercularis were no longer significantly negatively associated with *Basal Negative Affect*, and the hippocampal/amygdala complex was no longer significantly positively associated with *Positive Regulation*, suggesting these particular relationships between brain metrics and emotion components are a function of the effects of aging. The remaining specific brain–behavior pathways remained significant, indicating a unique contribution of individual differences in the volume of these brain regions to the emotion factors over and above the effects of age (Fig. 5, Fig. 6).

### Net emotional effects of age—results

Applying this approach, the relationship between older age and increased positive affectivity disappeared. The computed Net Positive Reactivity measure was negatively correlated with age (*r* = −0.188, *p* = 0.003) highlighting the sensitivity of scaling effects in measuring emotional responding. Further, there was no significant correlation between age and the computed Net Negative Reactivity (*r* = 0.071) or Net Negative Regulation (*r* = −0.006) measures. This highlights the importance of disaggregating positive and negative affectivity in the modeling to elucidate age-related positivity effects.

## Discussion

In a large population representative sample, we show that positive affect in response to emotional and neutral stimuli increases with age, even after controlling for behavioral and neural measures of declining executive control, in line with the positivity effect and the predictions of the SST (Carstensen, 1992, 2006) and ABM (Cacioppo et al., 2011). This was not the case for negative affect generated in response to negative stimuli, although we did find evidence of age-related increases in basal negative affect (based on negative affect ratings to neutral and positive stimuli). Furthermore, we show that emotion-related structural GM sources support and suppress affective responding and that certain brain–behavior relationships change with age.

A key aspect of the data was the breakdown of the net emotional effect often reported in studies of emotion regulation in older adults (Allard and Kensinger, 2014). Using this approach, we were able to separate positive and negative scales in the emotion regulation condition and show that *Positive Regulation* (deriving positive affect from negative material) significantly increased with age, whereas negative regulation (downregulating negative affect) did not. The data are thus in agreement with evidence of improved emotion regulation with age (Urry and Gross, 2010). Importantly, when using the computed net scores, the relationship between older age and increased positive affectivity disappeared. The computed net Positive Reactivity measure was negatively correlated with age, in line with our previous results (Schweizer et al., 2019). This highlights the importance of disaggregating positive and negative affectivity in the modeling to elucidate age-related positivity effects.

Additionally, we included structural gray matter sources derived from independent component analysis of structural MR data to examine brain–behavior relationships and the influence of age on those relationships. First, we observed decline in all gray matter sources associated with increasing age, in line with previous studies of age-related GMV loss (Kalpouzos et al., 2009). Using these sources, we then tested the diverging predictions of the amygdala-focused ABM and the frontal-focused cognitive control model at the neural level. We found no evidence in support of the ABM, with model comparison showing no unique contribution of the hippocampal/amygdala complex to any emotion factor and finding that age devalued the contribution of hippocampal/amygdala complex to *Positive Regulation*. Further, we found limited evidence for the cognitive control model, showing the pars opercularis of the IFG has a unique negative influence on *Basal Negative Affect* and *Positive Reactivity* as well as *Positive Regulation*. The IFG is a core emotion regulation brain region typically engaged when reappraising negative information and is implicated in reducing levels of negative affect (Goldin et al., 2008). However, our data suggest that the structural integrity of the IFG showed a negative relationship with several of our latent factors. One possibility is that the brain–behavior relationship between the IFG and *Positive Regulation* factor exerts a dampening effect on the positivity derived from negative stimuli. This is in line with our previous findings using the same stimuli in an imaging version of the task on a separate Cam-CAN sample, where decreased positivity was associated with decreased IFG activation (Schweizer et al., 2019). These effects could feasibly extend to the *Positive Reactivity* factor, whereby dispositional positivity is dampened as a function of spontaneous/automatic emotion regulation/control. Finally, we showed that age devalued the negative influence of the IFG and Medial FG on *Basal Negative Affect*, which speculatively may account for the unexpected observed increases in this factor score with age. Overall, and importantly in the context of the predictions of SST, age-related increases in *Positive Regulation* and *Positive Reactivity* remained significant over and above the negative influence of the neural measures.

In sum, this study used a population-derived sample from across the life span to investigate positive and negative affective responses to ecologically valid stimuli to explore the emotion/aging paradox. We found evidence broadly in line with the predictions of the SST, finding that positive responding increased with increasing age. Furthermore, these changes were related to the structural integrity of several neural regions typically associated with emotion regulation. We provide neural evidence against the aging brain model, instead showing that age devalues the contribution of the hippocampal/amygdala complex to *Positive Regulation*. Moreover, several of these brain–behavior relationships remain unaffected by age and may therefore constitute empirically derived neural markers to disentangle the paradox of increased well-being in old age.
